# Preliminary Characterization of Skin Microbiota and Mycobiota in Atopic Dermatitis by Metagenomic and Culture-Based Analyses

**DOI:** 10.3390/life16040690

**Published:** 2026-04-20

**Authors:** Federica Carraturo, Michela Salamone, Martina Annunziata, Eugenia Veronica Di Brizzi, Caterina Mariarosaria Giorgio, Arianna Petrillo, Ludovica Fedi, Angela Maione, Marco Guida, Emilia Galdiero

**Affiliations:** 1 Department of Biology, University of Naples Federico II, 80126 Naples, Italy; federica.carraturo@unina.it (F.C.); michela.salamone@unina.it (M.S.); martina.annunziata@unicampania.it (M.A.); marco.guida@unina.it (M.G.); emilia.galdiero@unina.it (E.G.); 2 Department of Experimental Medicine, University of Campania “Luigi Vanvitelli”, 80138 Naples, Italy; 3 Dermatology Unit, University of Campania “Luigi Vanvitelli”, 80138 Naples, Italy; eugeniaveronica.dibrizzi@gmail.com (E.V.D.B.); caterinagiorgio80@gmail.com (C.M.G.); 4 IRCCS Ca’ Granda Foundation, Ospedale Maggiore Policlinico, 20122 Milan, Italy; ariannapetrillo30@gmail.com; 5 Department of Translational Medical Science, Section of Pediatrics, University of Naples Federico II, 80131 Naples, Italy; ludovicafedi1@gmail.com

**Keywords:** atopic dermatitis, microbiota diversity, metagenomics, mycobiota, probiotics, culturomics

## Abstract

Atopic dermatitis (AD) is a chronic inflammatory skin disease influenced by several factors, including immune system imbalance, impairment of the epidermal barrier, and alterations in the composition of the gut and skin bacterial and fungal microbiota. This study combines metagenomic sequencing and culture-based methods to explore the impact of probiotic supplementation on the cutaneous microbiota and mycobiota of AD patients. Twenty-five adults diagnosed with AD were enrolled, and skin swabs were analyzed to characterize microbial diversity and load. Culturomic analyses identified 42 bacterial and 6 fungal species, confirming *Staphylococcus aureus* and *Candida parapsilosis* as predominant taxa. High-throughput sequencing revealed *Staphylococcus* spp. and *Malassezia* spp. as dominant genera, with notable interindividual variability. While probiotic use did not significantly influence bacterial diversity, it was associated with higher richness and evenness in fungal communities, as shown by alpha and beta diversity metrics. *Malassezia restricta* was more prevalent among probiotic users, whereas *Candida parapsilosis* and *Rhodotorula mucilaginosa* were enriched in non-users. These findings indicate an association between probiotic use and differences in the composition and diversity of the skin mycobiota compared with the bacterial microbiota, suggesting that fungal communities may be more responsive to probiotic-associated factors. Integrating metagenomic and culturomic approaches offers valuable insights into the complex interactions among host factors, microbial communities, and probiotic use in AD, paving the way for targeted microbiome-based therapeutic strategies.

## 1. Introduction

Atopic Dermatitis (AD) is a common, chronic, and recurring inflammatory skin disease, typically characterized by intense itching (pruritus), dry skin (xerosis), and eczematous lesions that vary with age. While in children the disease primarily affects the skin creases, in adults it often involves the head and neck, as well as chronic hand eczema, in addition to the classical presentation [1,2].

AD is often associated with other atopic conditions such as asthma and allergic rhinitis, and has a significant psychological and social impact. Patients may suffer from anxiety, depression, sleep disorders, ADHD, and reduced quality of life.

Although AD most commonly presents in early childhood, typically between 3 months and 2 years of age, it can persist into adulthood or, in some cases, first emerge later in life. While many children experience remission by the age of two, AD remains a prevalent condition, affecting an estimated 15–30% of children and 2–10% of adults. Notably, its prevalence has increased two- to threefold over the past three decades. AD is a multifactorial condition involving genetic predisposition, environmental triggers, and microbial influences. A key feature of the disease is skin barrier dysfunction, which increases vulnerability to microbial colonization and infection. There is growing evidence that the skin microbiome plays an essential role in the pathophysiology of AD. Indeed, the skin microbiome, typically composed of commensal and mutualistic microorganisms, can acquire pathogenic properties under conditions of immune dysregulation, an indicative trait of atopic dermatitis [3,4,5,6,7,8].

A substantial amount of research indicates that individuals with AD display distinct cutaneous microbiota phenotypes compared to healthy controls [9]. These microbial profiles appear to be dynamic and are influenced by both patient age and disease severity. Suwarsa et al. [10] identified significant variations in skin microbiota composition in AD patients stratified by disease severity, demonstrating that microbial diversity was notably reduced in individuals with moderate AD relative to those with milder forms of the disease. Similarly, Baochen et al. reported age-dependent differences in the skin microbiota of AD patients, with distinct microbial signatures observed among young children, adolescents, and adults, as determined through stratified microbiota analyses [11].

Alterations in the skin mycobiome have also gained recognition as critical contributors to the pathophysiology of various dermatological conditions. In the context of AD, fungal communities, particularly those dominated by *Malassezia* species, undergo marked compositional shifts. These changes promote immune dysregulation and cutaneous inflammation through mechanisms involving fungal allergens and enzymatic activities that compromise skin barrier integrity [12,13,14].

In addition to bacterial and fungal dysbiosis, recent advances in culturomics, the isolation and characterization of viable microorganisms through culture-based techniques, have underscored the importance of integrating data from the microbiome, mycobiome, and culturome. While high-throughput sequencing has greatly advanced the characterization of skin microbial communities, it provides limited information on microbial viability, functional activity, and strain-level phenotypic traits. In this context, culturomics complements sequencing-based approaches by enabling the isolation and quantitative assessment of live microorganisms, validating metagenomic findings, and facilitating functional investigations of host–microbe and microbe–microbe interactions. The integration of culture-dependent and culture-independent methods therefore offers a more comprehensive and biologically meaningful framework for studying cutaneous dysbiosis and its immunological implications in complex inflammatory conditions such as AD [15].

Despite these findings, current research has largely focused on isolated factors affecting the skin microbiota in AD, with limited investigation into the cumulative and interactive effects of multiple environmental and host-related variables. To date, large-scale studies assessing the combined impact of external factors, such as tobacco smoke exposure, comorbid conditions, dietary habits, and probiotic supplementation, remain lacking. Given the frequent alterations observed in both bacterial and fungal skin communities in patients with atopic dermatitis, driven by a complex interplay of host- and environment-related factors, there is a pressing need for comprehensive and integrative studies to elucidate how these variables collectively influence the composition and function of the cutaneous microbiome and mycobiome in the context of AD [16].

In particular, probiotic supplementation has recently emerged as a promising modulator of the gut–skin axis. However, its potential to reshape the cutaneous microbiota, especially the fungal microbiota, remains poorly understood. Preliminary evidence suggests that probiotics may exert indirect effects on skin microbial communities through immune regulation and barrier function, but direct impacts on fungal populations in AD have not been systematically studied. This knowledge gap underlines the importance of integrative investigations that simultaneously assess the microbiome, mycobiome, and culturome in relation to probiotic use. In this study, we explored whether oral probiotic intake might be associated with differences in the diversity and composition of the skin microbiota and mycobiota in patients with atopic dermatitis, by comparing individuals reporting regular probiotic use with those who did not, across different levels of disease severity. This investigation was motivated by the limited understanding of how systemic factors, such as dietary supplementation, may relate to the cutaneous microbial ecosystem in AD, particularly with respect to fungal communities, which remain underexplored compared to bacterial populations. By integrating metagenomic and culture-based approaches, the present study aims to provide preliminary, cross-kingdom insights into skin microbial dynamics and to identify potential microbial patterns associated with host and lifestyle characteristics, thereby addressing a relevant gap in current dermatological microbiome research.

## 2. Materials and Methods

### 2.1. Study Design and Participant Enrolment

We included 25 patients with AD from a cross-sectional, observational study within the Atopic Dermatitis (MetabAD) Cohort. Participants were recruited between January and July 2025 at the University Dermatology Clinic of Naples Vanvitelli, Italy. Eligible participants were over 18 years of age, had a history of AD for more than 10 years, and provided written informed consent.

Inclusion criteria were: age over 18 years, a confirmed diagnosis of AD based on the SCORAD index, presence of AD lesions localized to the arms, and no use of antifungal or antibiotic treatments within the 6 months preceding sample collection. Exclusion criteria included refusal to participate in the study or inability to provide informed consent.

The protocol for sample collection was reviewed and approved by the Ethics Committee of University Federico II of Naples (117/2023, 16 January 2024) and of the University of Campania Vanvitelli (15 July 2024 10.14-20240019440).

This study was conducted in full compliance with the ethical principles outlined in the Declaration of Helsinki, thereby ensuring respect for and the protection of all participants. Written informed consent was obtained from each participant prior to study initiation, permitting the publication of data derived from their personal test results. In order to maintain confidentiality, the results of this study were presented in a manner that anonymized the data, thereby preventing any possibility of identifying individual subjects.

### 2.2. AD Clinical Assessment

All patients underwent a full clinical assessment for AD. Disease severity was measured using the SCORAD (SCORing Atopic Dermatitis) score. Classification was made into mild, moderate or severe AD with a SCORAD score of <25, 25–50 or >50 respectively [17,18]. Data collected through a detailed questionnaire included information on: age and gender, smoking habit (smoker, non-smoker), recent use of probiotics (intake for at least three months), current pharmacological therapies, with particular attention to the use of biological drugs such as Dupilumab, presence of concomitant diseases [19], dietary habits [20] such as a Mediterranean, vegetarian, or vegan diet, and the presence of food intolerances such as gluten or lactose intolerance. This information was used to characterize the patient cohort and to investigate the correlation between clinical and environmental factors and the composition of the cutaneous bacterial and fungal microbiota.

### 2.3. Sample Collection and Culturomics of Skin Swabs

For the analysis of the fungal and bacterial microbial communities, skin swabs were collected from the participants’ skin [21].

The chosen sampling area was the skin of the arms, a region typically affected by the eczematous lesions of atopic dermatitis. For each patient, at least four sterile swabs (COPAN, Brescia, Italy) soaked in sterile 0.9% NaCl, were passed with a firm motion and by repeatedly rubbing over the affected area (surface area of 4 cm^2^) to adequately collect the superficial microorganisms [22]. The swabs were then transported to the laboratory under controlled temperature conditions, two from each patient were stored with 500 μL of lysis buffer (100 mM NaCl, 10 mM Tris-HCl [pH 8.0], 1 mM EDTA [pH 8.0], 1% sodium dodecyl sulfate, 2% Triton X-100) at −80 °C to preserve the genetic material and ensure the integrity of the samples until DNA extraction and subsequent Next-Generation Sequencing analysis. The other two swabs were employed for culture analysis. For quantitative microbiological analysis, 100 µL of swab preservation buffer was spread onto various selective and non-selective media after homogenizing the sample. Specifically, Tryptic Soy Agar (TSA, Oxoid, Basingstoke, UK) was used as a general-purpose medium for the isolation of aerobic mesophilic bacteria, Mannitol Salt Agar (MSA) for the selective isolation of staphylococci, and MacConkey agar (Sigma-Aldrich, St. Louis, MO, USA) for the isolation of Gram-negative bacteria. For fungal isolation, samples were plated on Sabouraud Dextrose Agar (SDA, Oxoid, Basingstoke, UK) supplemented with chloramphenicol (Oxoid, Basingstoke, UK) and on modified Dixon agar (Oxoid, Basingstoke, UK), a lipid-enriched medium suitable for the isolation of *Malassezia* spp. Plates were then incubated at 37 ± 1 °C for the detection of aerobic mesophilic bacteria and at 22 ± 1 °C for the isolation of yeasts and molds. Following microbial growth, approximately 300 colonies were isolated on the basis of a macroscopic analysis of the diverse colony morphologies. Isolated colonies were molecularly characterised, through Sanger Sequencing by an external service (Biofab Research srl, Rome, Italy), following the procedural steps described in our previous study [23]. Universal primers complementary to the conserved V3–V6 regions of the 16S rRNA gene for bacteria and ITS1-ITS4 complementary to the ITS-5.8S rDNA region of the fungal 18S rRNA gene [24] were used for the DNA amplification and subsequent Sanger sequencing assays.

### 2.4. Bacterial and Fungal Microbiota Analysis

Analysis of the bacterial and fungal microbiota profiles was conducted as described previously by our group [25], with slight modifications to the internal protocol for initial pretreatment to adapt the method to DNA extraction from swabs. Briefly, the swab was previously pretreated using glass beads, shaking constantly at 20 °C and 1500 rpm for 5 min. Each sample’s bacterial and fungal communities were investigated through Illumina MiSeq sequencing (2 × 300 bp, paired end; 600 cycles) according to the manufacturer’s instructions (Illumina MiSeq, San Diego, CA, USA), respectively targeting the 16S rRNA gene’s V3–V4 hypervariable region [26] and the Internal transcribed Spacer 2 (ITS2) [27]. Sequenced reads were processed with QIIME2 (https://qiime2.org/). Paired-end reads were demultiplexed and primers removed. The sequences were merged, quality checked using DADA2 and then counted and assigned to taxa using the Silva database (version 138.1; https://www.arb-silva.de/). Subsequent statistical analyses were performed with the XLSTAT add-in (Version 2014.5.03, Addinsoft, Paris, France) and R studio (RStudio version 2025.05.1+513, Posit Software, Boston, MA, USA, https://www.rstudio.com/) using the phyloseq, ggplot2, dplyr, scales, tidyverse, RColorBrewer, vegan, tidyr, ggpubr, cowplot, and ggrepel packages. Alpha Diversity indices were calculated (richness: Observed, Chao1, ACE; and evenness-diversity: Shannon, InvSimpson, Simpson). For Beta Diversity, the Bray–Curtis metric was used, with PERMANOVA tests and Non-metric Multidimensional Scaling (NMDS) followed by Differential Abundance Analysis (DESeq2). Bacterial and fungal relative abundances were examined at the genus and species levels.

## 3. Results

A total of 25 patients with AD were enrolled in the study, comprising 14 females and 11 males. As reported in Table 1, participants ranged in age from 18 to 80 years, with a mean age of 32 years. Overall, 32% of the subjects were smokers, and 48% were receiving biologic treatment with Dupilumab. Of the 25 patients, 36% were taking probiotics (strain composition, dosage, and duration were heterogeneous and not standardized), and this parameter was used to define the two study groups. Differences in the variability of the bacterial and fungal microbiota, and culturome were then evaluated between these groups in relation to changes in SCORAD, reflecting the severity of skin lesions.

Notably, there were no significant variations in SCORAD between the probiotic and non-probiotic groups.

### 3.1. Culturomics Analysis and Identification of Live Microbial Isolates

**Figure 1:**
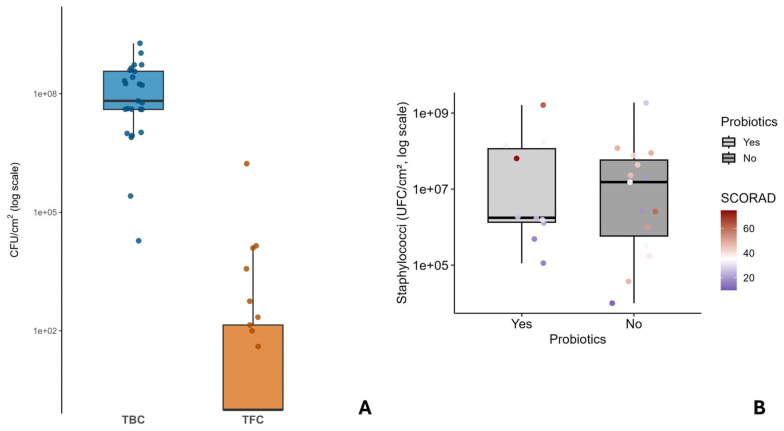
Comparative microbial load and its association with probiotic use and disease severity in patients with atopic dermatitis. (**A**) Comparative distribution of Total Bacterial Count (TBC) and Total Fungal Count (TFC). Box plots of CFU/cm^2^ (log scale) across 25 skin samples. Boxes indicate median and IQR; whiskers extend to non-outlier ranges; jittered points correspond to individual values. (**B**) Association between probiotic use, staphylococcal skin colonization and SCORAD index. Boxplots show the distribution of staphylococcal counts (UFC/cm^2^, log scale) in patients with and without probiotic use. Individual points represent single patients and are color-coded according to disease severity (SCORAD index).

Culturomic analyses were exploited to quantify the total microbial load on skin specimens and distinguish among bacterial and fungal components. The aggregate results are summarised in the box plot in Figure 1A, illustrating the distribution of microbial loads across all patients. Specifically, the microbial load is between 1 × 10^7^ and 1 × 10^8^ colony-forming units (CFU) per unit of skin surface area. Some outliers fall below or above this range, indicating variability between individuals in the study population. Conversely, the fungal load is ordinarily diminished by approximately four orders of magnitude, with mean values approximating 1 × 10^3^ CFU/cm^2^. The distribution of fungal load also exhibits variability; in more than half of the samples, no fungal growth was observed and only one sample showed a fungal load of around 10^6^ CFU/cm^2^.

Non-targeted bacterial and fungal isolation was performed to determine the role that different strains might play in atopic dermatitis. Approximately 300 isolates were obtained from the 25 swabs, using different culture media to obtain a higher microbial variability. Sanger sequencing of the 16S rRNA and ITS rRNA genes allowed the isolates to be taxonomically characterized, resulting in the classification of 42 different bacterial and 6 fungal species (Table S1).

Culturomics analysis performed on selective media allowed the quantification of the *Staphylococcus* spp. load. Comparison with total bacterial load showed that the values were comparable in some subjects. This allows us to hypothesise that this bacterial community represents the predominant component of the skin microbiota. The staphylococcal load was further correlated with the SCORAD score, reflecting the clinical severity of atopic dermatitis, and with probiotic use, as shown in Figure 1B.

The staphylococcal load varies by several orders of magnitude, ranging from ~10^5^ to ~10^9^ CFU/cm^2^. The box plot displays a lower median in the group of patients regularly using probiotics in comparison to those not assuming the nutritional supplement.

Individual data points are superimposed on the box plots, color-coded according to the SCORAD (SCORing Atopic Dermatitis) index: dark red indicates a higher SCORAD score (more severe atopic dermatitis, up to ca. 80); light/white indicates an intermediate SCORAD score (ca. 40); dark purple indicates a lower SCORAD score (less severe atopic dermatitis, down to ca. 0).

The plot visually suggests that the median Staphylococci concentration appears lower in the “yes” (Probiotics) group approximately 3 × 10^6^ UFC/cm^2^) compared to the “no” (no Probiotics) group (approximately 1.5 × 10^7^ UFC/cm^2^). Thus, higher staphylococcal load values are generally associated with higher scores, suggesting a positive correlation between clinical severity and bacterial density. However, the Mann–Whitney test revealed no statistically significant differences between patients using probiotics and those not (W = 71, *p* = 0.85). Although the boxplot shows a tendency towards lower median bacterial load values in the first group of patients, intra-group variability and the limited sample size do not allow definitive conclusions to be drawn.

### 3.2. Skin Microbiota/Mycobiota Across Probiotics Assumption

**Figure 2:**
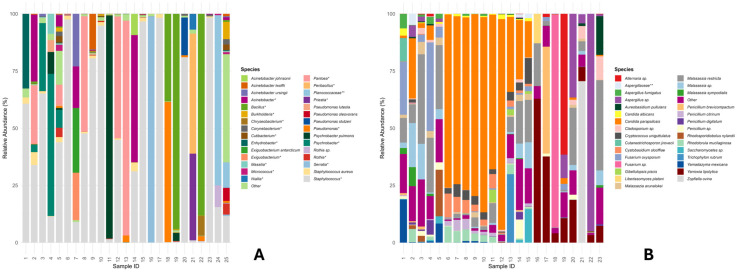
Relative abundance of bacterial and fungal taxa. (**A**) Relative abundance of bacterial taxa identified by 16S rRNA sequencing. (**B**) Relative abundance of fungal taxa identified by ITS1 sequencing. Stacked bar plots display the relative abundance of species across samples; colors denote distinct taxa. * (Genus level), ** (Family level).

Figure 2A shows a species-level barplot highlighting an uneven distribution of skin microbiota among different subjects. The bacterial community is dominated by *Staphylococcus* spp., which accounted for approximately 90% of the relative abundance in many specimens. Lower abundances of *Staphylococcus aureus* were detected, ranging from 0.5% to 5% in more than half of the analysed cohort. The genus *Pantoea* also was highly abundant, accounting for up to 50% of the total microbial community in some individuals. The genera *Psychrobacter* and *Acinetobacter* are also well represented, identified at the species level as *A. johnsonii*, *A. lwoffii*, and *A. ursingii*. As for the genus *Psychrobacter*, *P. pulmonis* represents 98% of the microbial community in sample 11.

The analysis of fungal DNA using the ITS1 molecular target resulted in no amplification for 24 and 25 swab samples, yielding insufficient fungal DNA for sequencing. Therefore, these samples were excluded from subsequent comparative analyses. Some subjects showed profiles dominated almost exclusively by a single fungal species (e.g., *Malassezia restricta* or *Candida parapsilosis*), while others were characterised by substantial diversity. Figure 2B only shows the 30 most abundant species (the remaining species were summed as “Other”). *Candida parapsilosis* was reported with a very low relative abundance in some samples and was not detected in two samples; in others, it resulted present with a relative abundance of 92% (e.g., sample 12). *Malassezia restricta* is present in all samples, with relative abundances ranging from 0.5% to 40% (e.g., sample 23). *Yarrowia lipolytica* is also very well represented, being highly abundant in samples 16 to 23. *Candida albicans*, on the other hand, is present at low abundances ranging from 0.3% to 3%. *Cystobasidium slooffiae* is present in approximately 60% of samples, with relative abundances ranging from 0.1% to 10%. Other species of the *Malassezia* genus are also present, including *M. arunalokei*, *M. sympodialis* and *Malassezia* sp., which are among the most abundant. Several species of the *Penicillium* genus are also present, including *P. brevicompactum*, *P. citrinum* and *P. digitatum*. However, only *P. brevicompactum* was retrieved with an approximate relative abundance of 50% in sample 17 (Figure 2B).

In the bacterial community, as shown in Table S2, none of the indices revealed statistically significant differences for the parameter “Use of Probiotics”. By contrast, in the fungal community, as demonstrated in Table S3, all Alpha Diversity indices tested for this parameter (richness: Observed, Chao1, and ACE; evenness/diversity: Shannon, InvSimpson, and Simpson) show a *p*-value lower than 0.05. The statistical results indicate a statistically significant difference in both richness and evenness/diversity in the Alpha Diversity of the skin fungal microbiome between patients receiving probiotics and those not receiving probiotics.

Based on the values of the indices that provide information on diversity (Shannon, Simpson, and InvSimpson), i.e., the number of species and how evenly they are distributed, it can be inferred that probiotic intake has a strong impact on the structure and balance of fungal species on the skin, with greater diversity observed in those who take them. The other three indices show that observed richness is also statistically influenced by this parameter.

For beta diversity analysis the NMDS graph (Figure S2A) shows a good two-dimensional representation of the bacterial community data, with a stress value of less than 0.2 (considered acceptable). However, the PERMANOVA analysis (b) does not show any statistically significant differences (R^2^ = 0.055, *p*-value = 0.196), suggesting that the variability in the bacterial community is not attributable to the tested factor. For the fungal community (Figure S2B) the NMDS plot provides a faithful representation of the original data, with low stress (0.1) in two dimensions. Probiotic intake explains approximately 18% of the total variation in microbial composition, and this is statistically significant. The samples from the two groups form distinct clusters, confirming that probiotic intake has altered the beta diversity of the fungal community in a non-random way. However, when a differential abundance analysis (DAA) is performed, most microorganisms do not exhibit a significant or statistically robust change in abundance in response to probiotic treatment, with the exception of the fungus *Cystobasidium slooffiae*. This analysis revealed that the abundance of several bacterial taxa is significantly associated with probiotic use (*p* < 0.05). *Pseudomonas luteola* and the genus *Psychobacter* manifest greater prevalence in subjects ingesting probiotics. In subjects who do not use probiotics, a significant reduction (negative log2 fold change) has been shown by several bacterial taxa; these include Hydrogenophaga, Planococcaceae, *Acinetobacter lwoffii*, *Paracoccus* sp., and Rothia sp. The resulting Volcano Plot are shown in the Supplementary Materials (Figures S4 and S5).

**Figure 3:**
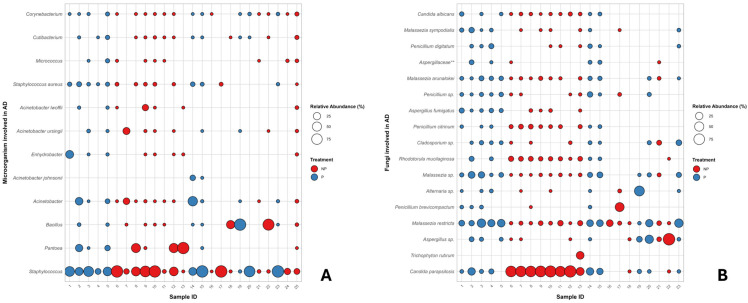
Bubble chart of bacterial 16S rRNA (**A**) and ITS1 rRNA (**B**) gene profiles at the species level. Coloured circles indicate probiotic use (P, blue) or non-use (NP, red) in relation to bacterial/fungal species typically detected in the skin microbiome of patients with atopic dermatitis (AD). Bubble size reflects relative abundance. ** (Family level).

Distinct mycobiota and microbiota profiles were observed depending on the sample type and on whether the samples originated from patients receiving probiotics or from control subjects. To further explore these differences, a correlation analysis was performed and visualized in a bubble plot. This analysis examined bacterial and fungal (Figure 3) species previously associated, according to the literature, with skin dysbiosis, and assessed their relationship with probiotic intake.

Correlation analysis (available in Figure 3A) highlighted a prevalence of the genera *Staphylococcus*, *Pantoea*, and *Bacillus* [28,29]. The selected taxa demonstrate significant interindividual variability, with the initial genus present in nearly all samples, yet exhibiting both elevated and diminished relative abundances in subjects using or not probiotics. *Pantoea* sp. is more associated with patients not using probiotics; likewise, *Acinetobacter* and related species, though underlining lower relative abundances in untreated (i.e., NP) than in treated subjects (i.e., P). The presence of skin commensals, such as *Cutibacterium* and *Corynebacterium* species, is consistent across different subjects. The use of probiotics is generally linked to a drop in the number of opportunistic bacteria and an increase in commensal taxa. Figure 3B’s *Y*-axis lists the different fungal species or genera identified in the samples (e.g., *Candida albicans*, *Malassezia sympodialis*, *Aspergillus* sp., *Trichophyton rubrum*); the *X*-axis represents the 23 individual samples analyzed. The plot allows for the identification of pervasive and dominant species. Dominant species like *Malassezia sympodialis*, *Aspergillus* sp., and *Penicillium citrinum,* are present (small to medium dots) in the majority of the samples (both red and blue). while dominant colonizing species (i.e., whith high relative abundance) are mostly 3, characterized in specific samples, notably: *Candida parapsilosis*, highly abundant (large red circles) primarily in samples 6 through 13; *Trichophyton rubrum*, mainly in samples 21 and 22; and *Alternaria* sp., showing a high abundance (large blue circle) in sample 19.

The distribution of the species based on the probiotics administration variable (No Probiotics, red; Probiotics, blue) appears varied. Some species (e.g., *Candida albicans*, *Malassezia sympodialis*) are found across most samples regardless of the probiotics assumption, while others show high abundance primarily linked to the non-assumption of probiotics. In particular, correlation analysis shows that the relative abundance of *Candida parapsilosis,* as much as *Rhodotorula mucilaginosa* is significantly higher in patients who do not use probiotics, while the opposite trend is observed for *Malassezia restricta* and other species belonging to the *Malassezia* genus, which are more prevalent in subjects who use probiotics.

Species belonging to the genus *Aspergillus* show generally low abundances in all samples, with the exception of sample 22, which relates to a patient who does not use probiotics.

It is interesting to observe the overall composition of the microbial and fungal communities of some patients, as this can provide insights into their health and well-being. For example, among subjects who do not take probiotics, patient 12 has a skin bacterial community consisting almost exclusively of *Staphylococcus* spp. and *Pantoea* spp., while the fungal community is dominated by *Candida parapsilosis* with the marginal presence of a few other genera. In general, microbial communities appear less variable in patients who do not use probiotics; on the contrary, in patient 1, who takes probiotics, the fungal community shows a much more heterogeneous composition, with at least 15 genera present in significant relative abundances.

Principal coordinates analysis (PCoA), which is based on the Bray–Curtis dissimilarity index, was used to evaluate the differences in microbial composition between the two groups of samples.

**Figure 4:**
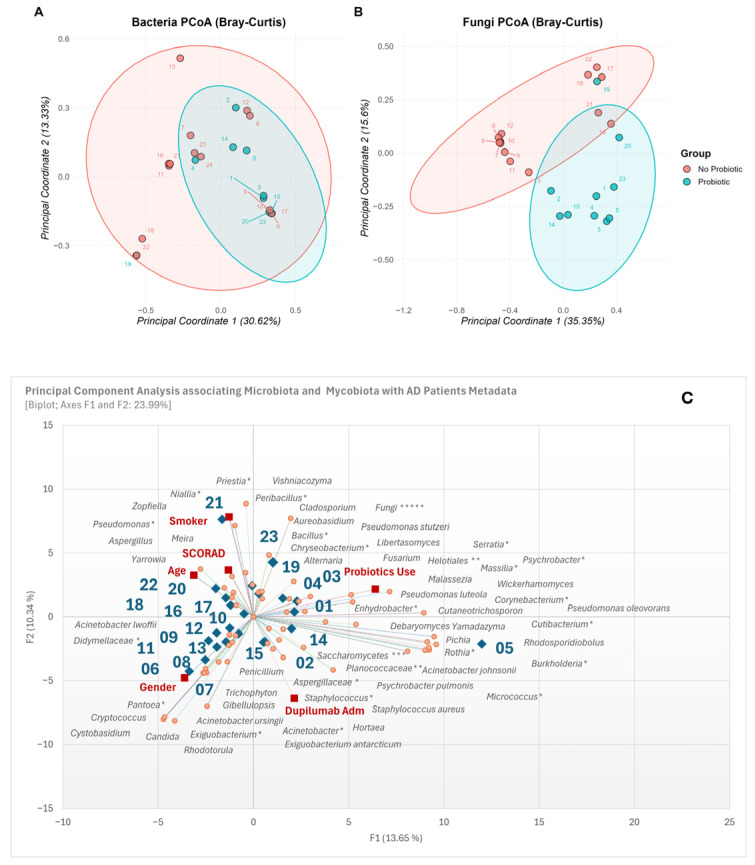
A Principal Coordinate Analysis (PCoA) of microbial community structure was performed based on Bray–Curtis dissimilarity, taking into account the division of patients into two groups: those who use probiotics and those who do not. Panel (**A**) shows bacterial communities, and panel (**B**) shows fungal communities. (**C**) Principal component analysis (PCA) ordination diagram showing the distribution of the plots along two axes based on all metadata. The blue labels (numbers) indicates the individual patients, the red labels indicate the clinical and demographic variables, black labels and orange dots correspond to microbial taxa (microbiota and Mycobiota variables); * (Genus level), ** (Family level), *** (Order level), ***** (Phylum level). Principal components (PCs) axes: PC1 = 13.65% and PC2 = 10.34% of the total variance explained.

Almost 50% of the total variability observed between samples can be explained by the first two principal axes both for the fungal and bacterial communities. Analysis of the bacterial community (Figure 4A) shows no division between the two groups, even though samples from patients taking probiotics appear to be clustered together (PERMANOVA, F = 1.35, R^2^ = 0.055, *p* = 0.177). Using probiotics explains around 5.5% of the total variation in skin bacterial community composition.

On the other hand, in the fungal communities (Figure 4B) the analysis shows a significant separation between the two groups (*p* < 0.05), suggesting overall differences in microbial composition associated with probiotic assumption. However, there are some individual exceptions to this. For example, patient 19 has a composition more similar to that of subjects who do use probiotics, compared to those not using the supplements. Patients 21 and 16 are positioned in the intersection area between the two groups, indicating intermediate microbial characteristics.

Principal Component Analysis results in Figure 4C highlight the distribution of samples (blue rhombus), clinical and demographic metadata (red square), and the most abundant bacterial and fungal ASVs (orange dots).

All available metadata were included in the PCA to explore the factors contributing to variation in the skin bacterial and fungal communities. The biplot allows for the interpretation of correlations based on vector direction and proximity. The SCORAD and Age metadata variables are tightly clustered and pointing towards the negative F1 and negative F2 quadrant, suggesting a strong positive correlation between higher disease severity and older age in this cohort. Moreover, fungal genera such as *Meira* and *Aspergillus*, and the bacteria belonging to *Pseudomonas* and *Yarrowia* genera are located near the SCORAD/Age cluster, implying these taxa are positively associated with older patients and higher AD severity. The Probiotics Use vector points towards the positive F1 and positive F2 quadrant, associating this factor with samples like 01, 02, 04 and 19. Fungal genera like *Malassezia*, *Pichia*, and *Rhodosporidiobolus* are positioned nearby, potentially indicating a higher prevalence of these taxa in patients using probiotics, as much as bacterial species belonging to *Enhydrobacter*, *Pseudomonas* and *Chryseobacterium* genera. The Dupilumab Administration vector points towards the negative F2 and positive F1 quadrant. This factor is substantially associated with samples 02, 14 and 15, with fungal genera such as *Saccharomycetes*, *Pichia*, and *Cryptococcus*, and species belonging to *Staphylococcus* (e.g., *S. aureus*), *Acinetobacter* (e.g., *A. ursingii*), and *Exiguobacterium* genera. Based on the gender, males are more frequently associated with the genera *Candida*, *Rhodotorula*, *Cystobasidium, Cryptococcus* and *Pantoea.* Conversely, smokers show a stronger correlation with the genera *Aspergillus*, *Meira*, *Yarrowia*, *Zopfiella*, *Pseudomonas* and *Peribacillus*, suggesting that the smoking habit might affect the modulation of the skin micro- and myco-biota. The *Malassezia, Fusarium, Chryseobacterium,* and *Serratia* mainly highlight a positive correlation with Probiotics use: the outcome suggests that individuals who generally use probiotics present a more diversified and heterogeneous microbial composition than those not supplemented with probiotics. Overall, the PCA biplot effectively distinguishes patient clusters based on their bacterial and fungal profiles and reveals distinct relationships between specific micro- and myco-biota categories and key clinical parameters (SCORAD, Age), supplementation (Probiotics) and interventions (Dupilumab).

## 4. Discussion

The global incidence of AD has risen significantly in recent years, contributing to substantial economic, emotional, and social burdens for affected individuals and their families. Although genetic predisposition, environmental exposures, immune dysregulation, and impaired skin barrier function are known contributors to AD pathogenesis, the underlying mechanisms remain only partially understood, and current therapeutic strategies often provide limited or inconsistent results [30].

A growing body of evidence highlights the critical role of the skin microbiota in maintaining epidermal homeostasis, regulating immune responses, and preserving barrier integrity. Alterations in microbial composition, both in diversity and functional activity, are strongly implicated in the pathogenesis of chronic inflammatory skin diseases such as atopic dermatitis, psoriasis, acne vulgaris, and rosacea. Dysbiosis is now considered not merely a consequence of skin inflammation but an active driver of disease severity, capable of shaping clinical manifestations through shifts in key bacterial and fungal communities [31,32].

Dietary habits have also been linked to AD risk. Diets rich in fiber and probiotics have been associated with reduced atopic dermatitis incidence, supporting the hypothesis that nutritional modulation may influence immune responses and gut microbiota dynamics [33,34,35,36]. This connection is in line with the aim of the present study, which investigates the relationship among probiotics, skin microbiota and mycobiota, and AD severity, offering preliminary insights into the probiotics–skin microbiota/mycobiota–AD axis [37,38,39]. In this study, we explored whether differences in the composition of the skin microbiota and mycobiota could be observed in patients with AD across varying clinical and demographic characteristics, including reported probiotic use, by integrating culture-based (culturomic) and metagenomic approaches. Importantly, this study was designed as a preliminary cross-sectional observational analysis and not as a controlled interventional trial. Therefore, the comparisons performed between patients reporting probiotic use and those not reporting it should be interpreted as exploratory associations rather than evidence of causality or treatment effect. This combined strategy enabled both quantitative assessment of viable microorganisms and high-resolution profiling of microbial diversity [40]. Culturomic analyses revealed a predominance of bacterial colonization compared to fungi, with an overall microbial load of approximately 1 × 10^8^ CFU/cm^2^ for bacteria and 1 × 10^3^ CFU/cm^2^ for fungi. *Staphylococcus* species represented the dominant bacterial taxa, consistent with previous reports implicating *Staphylococcus*, particularly *S. aureus*, in AD pathogenesis and disease severity. In contrast, cultivable fungal communities were less abundant and more variable, reflecting both biological heterogeneity and the limitations of culture-based methods for fungal detection. High-throughput sequencing substantially expanded the resolution of microbial profiling, identifying a broader spectrum of bacterial and fungal taxa, including *Staphylococcus aureus* and *Candida parapsilosis* as prominent species within their respective kingdoms. Notably, metagenomic analysis of the ITS region revealed a complex and diverse mycobiome that was only partially captured by culture-based approaches, underscoring the importance of integrating molecular and cultivation techniques to obtain a comprehensive view of skin microbial ecosystems. When associations between microbial composition and clinical or lifestyle variables were examined, no significant relationships were observed between bacterial diversity and factors such as age, gender, smoking status, or therapeutic history. Similarly, reported probiotic use did not significantly affect bacterial alpha diversity, suggesting that any potential effects on bacterial communities may be indirect or context-dependent. In contrast, fungal communities exhibited significantly higher richness and evenness in individuals reporting probiotic use, as indicated by multiple alpha diversity metrics. Beta diversity analyses further supported these findings, revealing distinct fungal community structures associated with this variable, whereas bacterial community structure remained largely unchanged.

This observation suggests that probiotic use may be associated with changes in the skin environment that correlate with differences in fungal populations. Nevertheless, this interpretation should be made with caution since probiotic intake was self-reported and no longitudinal sampling before and after supplementation was available. As a consequence, the present design does not allow direct assessment of temporal microbiota changes attributable to probiotic exposure.

Of particular interest was the association between probiotic use and increased abundance of *Cystobasidium slooffiae*, which may indicate that specific fungal taxa could potentially serve as biomarkers associated with probiotic use.

Similar cross-kingdom interactions have been described in the gut, where probiotic supplementation can improve symptoms of functional bowel disorders by restoring microbial balance [41]. Indeed, previous studies demonstrate that probiotics can increase beneficial taxa such as *Faecalibacterium prausnitzii* and *Blautia stercoris*, and influence metabolite profiles tied to gut–brain axis regulation, supporting the broader concept that probiotics exert multisystem effects through microbiome modulation [42].

In our skin samples, *Staphylococcus*, *Pantoea*, and *Bacillus* were among the most prevalent bacterial genera. Opportunistic taxa such as *Pantoea* and *Acinetobacter* were more frequent in subjects not consuming probiotics, whereas commensals such as *Cutibacterium* and *Corynebacterium* were consistently present across groups. Probiotic use generally correlated with reduced abundance of opportunistic bacteria and increased representation of commensal taxa, suggesting a shift towards a healthier microbial profile.

Fungal communities also differed notably with probiotic intake. While *Candida albicans* and *Malassezia sympodialis* were widespread across all groups, *Candida parapsilosis* and *Rhodotorula mucilaginosa* were enriched in non-probiotic users. Conversely, *Malassezia restricta* and other *Malassezia* species were more abundant in probiotic users. *Aspergillus* spp. were generally rare, appearing prominently in only one non-probiotic sample. The finding that *Malassezia* spp. were significantly reduced in AD subjects not using probiotics, together with a corresponding increase in *Candida* colonization, is consistent with previous studies describing a shift from commensal to opportunistic fungi in dysbiosis-associated inflammatory skin states [43,44].

Our results are also in agreement with studies [45] showing that topical probiotic formulations can modulate the cutaneous microbiome and mycobiome, decreasing *Staphylococcus* species and altering fungal–bacterial interactions, particularly within the *Malassezia* genus. Healthy skin mycobiota is typically dominated by *Malasseziaceae*; in AD, however, *M. globosa* abundance decreases while *M. dermatis* and *M. sympodialis* increase. In our cohort, *M. globosa* was only detected in one probiotic-using subject, whereas *M. sympodialis* was detected in both groups but was slightly more common among non-probiotic users [46].

Several limitations of this study should be acknowledged. First, the cohort size was relatively small and clinically heterogeneous, including variability in age, disease severity, and treatment exposure, which may have reduced statistical power and limited the generalizability of the findings. Second, the absence of a healthy control group prevented discrimination between microbial features specifically associated with AD and those potentially related to other host- or lifestyle-related factors. Although probiotic use was associated with measurable alterations in fungal community composition, these changes were not accompanied by clinically meaningful improvements in AD severity in the present study. Accordingly, the observed variations in fungal diversity should be regarded as preliminary and descriptive, and caution is warranted when considering any potential clinical implications. Nonetheless, alterations in microbial composition may still hold clinical significance in larger cohorts or longitudinal studies. However, due to the absence of longitudinal follow-up, it was not possible to assess temporal changes in microbial communities or to evaluate the long-term effects of external factors, including probiotic intake or pharmacological treatments, on skin microbiota and mycobiota composition. In addition, because probiotic use was not evaluated within a controlled pre-/post-intervention design, the study cannot determine whether the observed microbial differences preceded probiotic intake or emerged subsequently. Accordingly, the present findings should be considered descriptive and hypothesis-generating. Integrating metagenomic data with functional analyses, such as fungal metabolite profiling and cross-kingdom interaction studies, will be crucial for clarifying the mechanistic role of microbial communities in AD pathophysiology. The combination of culture-based and molecular methods offers valuable translational potential, enabling improved diagnostic assessment of microbial dysbiosis and informing future research aimed at understanding whether targeted microbiome-based interventions could be developed.

In conclusion, although this study is limited by a small sample size, uneven demographic distribution, population heterogeneity, the absence of healthy controls, and the lack of longitudinal pre-/post-probiotic sampling, the integrated metagenomic and culturomic approach demonstrated its value in characterizing dysbiosis associated with atopic dermatitis. Expanding this framework in future research will be crucial to clarify the interactions between beneficial and pathogenic microbial colonizers and to evaluate the potential role of probiotic supplementation in modulating the skin microbiota and mycobiota of AD patients. In addition, longitudinal studies incorporating repeated sampling will be necessary to capture temporal changes in skin microbial communities and to establish whether microbial shifts occur prior to or as a consequence of disease severity. Future studies based on larger and more homogeneous cohorts, including healthy controls and repeated sampling before and after probiotic administration, will be essential to validate these preliminary observations and to clarify whether microbial shifts are associated with disease severity, probiotic exposure, or both.

## Figures and Tables

**Table 1:** Baseline demographics and disease characteristics. Age is expressed as median (range). SCORAD is AD area severity index.

Sample ID	Gender	Age	Smoker	Probiotics Supplementation	Dupilumab Administration	SCORAD
01SW	F	47	Yes	Yes	Yes	21.6
02SW	F	24	No	Yes	Yes	31.4
03SW	M	33	No	Yes	No	26.3
04SW	M	72	No	Yes	No	14.9
05SW	M	30	No	Yes	Yes	25.7
06SW	F	48	No	No	Yes	41.2
07SW	F	80	No	No	Yes	19.6
08SW	F	68	No	No	No	45.2
09SW	F	64	Yes	No	No	49
10SW	F	25	Yes	No	No	35.1
11SW	F	19	No	No	No	33.5
12SW	M	18	No	No	Yes	51.6
13SW	M	40	No	No	Yes	39
14SW	M	19	No	Yes	Yes	33.1
15SW	F	18	No	Yes	Yes	37.4
16SW	M	32	No	No	Yes	47.8
17SW	M	60	No	No	Yes	26.7
18SW	M	34	No	No	No	18.6
19SW	F	22	Yes	Yes	No	15.4
20SW	F	79	Yes	Yes	No	74.5
21SW	M	78	Yes	No	No	43.5
22SW	F	62	Yes	No	No	61
23SW	M	31	Yes	Yes	No	64
24SW	F	67	Yes	No	No	49.6
25SW	F	21	No	No	No	10

## Data Availability

The original contributions presented in this study are included in the article/Supplementary Materials. Further inquiries can be directed to the corresponding author.

## Supplementary Materials

The following supporting information can be downloaded at https://www.mdpi.com/article/10.3390/life16040690/s1, Table S1. Molecular identification of bacterial and fungi isolates from skin swabs of patients with atopic dermatitis (AD) performed using Sanger sequencing. The table lists the “Sample code” corresponding to the swabs in which each microorganism was detected, along with the species’ characteristics and their reported associations with skin diseases and/or atopic dermatitis. The codes in the table refer to: P—Pathogen, O—Opportunistic, N—Non-pathogen/Saprophytic, NE—No evidence. Table S2. Results of the statistical analysis for the evaluation of alpha-diversity in the bacterial community, conducted using the Wilcoxon rank sum test, with indications of *p*-values (statistically significant differences between the samples compared are indicated in bold) and W-statistics. Table S3. Results of the statistical analysis for the evaluation of alpha-diversity in the fungal community, conducted using the Wilcoxon rank sum test, with indications of *p*-values (statistically significant differences between the samples compared are indicated in bold) and W-statistics. Figure S1. Alpha diversity calculated using the Shannon diversity index and the Chao1 richness estimator for both bacterial and fungal communities in subjects either using probiotics (“yes”) or not (“no”). Specifically: (A) bacterial Shannon index, (B) bacterial Chao1 richness, (C) fungal Shannon index, and (D) fungal Chao1 richness. Statistical differences between groups were assessed using the Wilcoxon rank-sum test. Figure S2. Beta diversity visualized using a Non-metric Multidimensional Scaling (NMDS) plot based on Bray–Curtis dissimilarity in subjects either using probiotics (“yes”) or not (“no”). Panel (A) shows bacterial communities, and panel (B) shows fungal communities. Statistical differences between groups were assessed using the PERMANOVA test. Figure S3. Differential abundance (DESeq2) of bacterial taxa between probiotic users (“yes”) and non-users (“no”), showing taxa significantly enriched (green) or decreased (red) with probiotic use (*p* < 0.05). Figure S4. Volcano plot of bacterial taxa differentially abundant between probiotic users and non-users (*p* < 0.05). Figure S5. Volcano plot of fungal taxa differentially abundant between probiotic users and non-users (*p* < 0.05).
