# Supplementary material for: Preliminary Characterization of Skin Microbiota and Mycobiota in Atopic Dermatitis by Metagenomic and Culture-Based Analyses
Source: Life (Basel). 2026 Apr 20;16(4):690. doi: 10.3390/life16040690 (PMC13117316; doi:10.3390/life16040690)
Supplement: Supplementary file 1 [file life-16-00690-s001.zip › life-4185599-supplementary.pdf]

Supplementary Information

Table S1. Molecular identification of bacterial and fungi isolates from skin swabs of patients with atopic dermatitis (AD) performed using Sanger sequencing. The table lists the "Sample code" corresponding to the swabs in which each microorganism was detected, along with the species' characteristics and their reported associations with skin diseases and/or atopic dermatitis. The codes in the table refer to: **P** – Pathogen, **O** – Opportunistic, **N** – Non-pathogen/Saprophytic, **NE** – No evidence.

Sample ID	Microorganism	Pathogenicity characteristic	Associated to AD and/or skin dysbiosis	Reference(s)
**01**	*Acinetobacter lwoffii*	O	X	[1]
**07**	*Acinetobacter septicus*	O		[2]
**12; 19**	*Aerococcus urinaeequi*	O		[3]
**12; 19**	*Aerococcus viridans*	O		[3]
**04; 22**	*Bacillus cereus*	O	X	[4]
**02**	*Bacillus paralicheniformis*	O		[5]
**03**	*Brucella anthropi*	O	X	[6]
**03**	*Cytobacillus solani*	O		[7]
**06; 07; 09**	*Enterococcus faecalis*	O		[8]
**13**	*Erwinia soli*	N		[9]
**02**	*Exiguobacterium acetylicum*	P		[3]
**02**	*Exiguobacterium indicum*	NE		[10]
**07**	*Exiguobacterium undae*	NE		[11]
**16**	*Klebsiella michiganensis*	O		[12]
**16**	*Klebsiella pasteurii*	O	X	[13]
**07**	*Mammaliicoccus sciuri*	O	X	[14]
**24**	*Micrococcus luteus*	N	X	[15]
**21**	*Niallia nealsonii*	NE		[16]
**03**	*Pantoea agglomerans*	O		[17]

**21**	*Peribacillus frigoritolerans*	N		[18]
**24**	*Rothia terrae*	NE		[19]
**05**	*Sphingomonas zeae*	O		[20]
**02; 03; 04; 05; 10; 14; 17**	*Staphylococcus aureus*	O	X	[21]
**25**	*Staphylococcus capitis*	O	X	[22]
**25**	*Staphylococcus capitis subsp. urealyticus*	O	X	[23]
**24**	*Staphylococcus caprae*	O	X	[24]
**11; 16; 18; 20; 22; 24; 25**	*Staphylococcus epidermidis*	O	X	[25]
**14; 23**	*Staphylococcus equorum*	N		[26]
**23**	*Staphylococcus equorum subsp. equorum*	N		[27]
**22**	*Staphylococcus equorum subsp. linens*	N		[28]
**08; 19; 20**	*Staphylococcus haemolyticus*	O	X	[29]
**20; 22; 25**	*Staphylococcus hominis*	O	X	[30]
**16**	*Staphylococcus lugdunensis*	O	X	[31]
**21; 24**	*Staphylococcus pasteuri*	NE		[32]
**07; 13**	*Staphylococcus saprophyticus*	O		[33]

**15**	*Staphylococcus schweitzeri*	O		[34]
**07**	*Staphylococcus vitulinus*	O		[35]
**10; 12; 16; 19; 21; 22**	*Staphylococcus warneri*	O	X	[36]
**07**	*Staphylococcus xylosus*	O		[37]
**03; 18; 19**	*Stenotrophomonas maltophilia*	O	X	[38]
**20**	*Streptococcus salivarius*	O		[39]
**20**	*Stutzerimonas stutzeri*	O	X	[40]
**02; 14**	*Candida parapsilosis*	O	X	[41]
**05**	*Filobasidium magnum*	P		[42]
**02; 07**	*Filobasidium uniguttulatum*	O	X	[43]
**08**	*Gibellulopsis nigrescens*	N		
**13**	*Naganishia diffluens*	P	X	[44]
**02; 11**	*Rhodotorula mucilaginosa*	O	X	[45]

Table S2. Results of the statistical analysis for the evaluation of alpha-diversity in the bacterial community, conducted using the Wilcoxon rank sum test, with indications of p-values (statistically significant differences between the samples compared are indicated in bold) and W-statistics.

Method	Index	W-Statistic	p-value
**Wilcoxon rank sum test with continuity correction**	Observed	80	0,8026
	Chao1	80	0,8026
	Shannon	55	0,2794
	InvSimpson	57	0,3316
	ACE	78	0,8896
	Simpson	57	0,3316

Table S3. Results of the statistical analysis for the evaluation of alpha-diversity in the fungal community, conducted using the Wilcoxon rank sum test, with indications of p-values (statistically significant differences between the samples compared are indicated in bold) and W-statistics.

Method	Index	W-Statistic	p-value
**Wilcoxon rank sum test with continuity correction**	Observed	8	**0,0004**
	Chao1	11	**0,0009**
	Shannon	4	**0,0001**
	InvSimpson	5	**0,0002**
	ACE	15	**0,0054**
	Simpson	5	**0,0002**

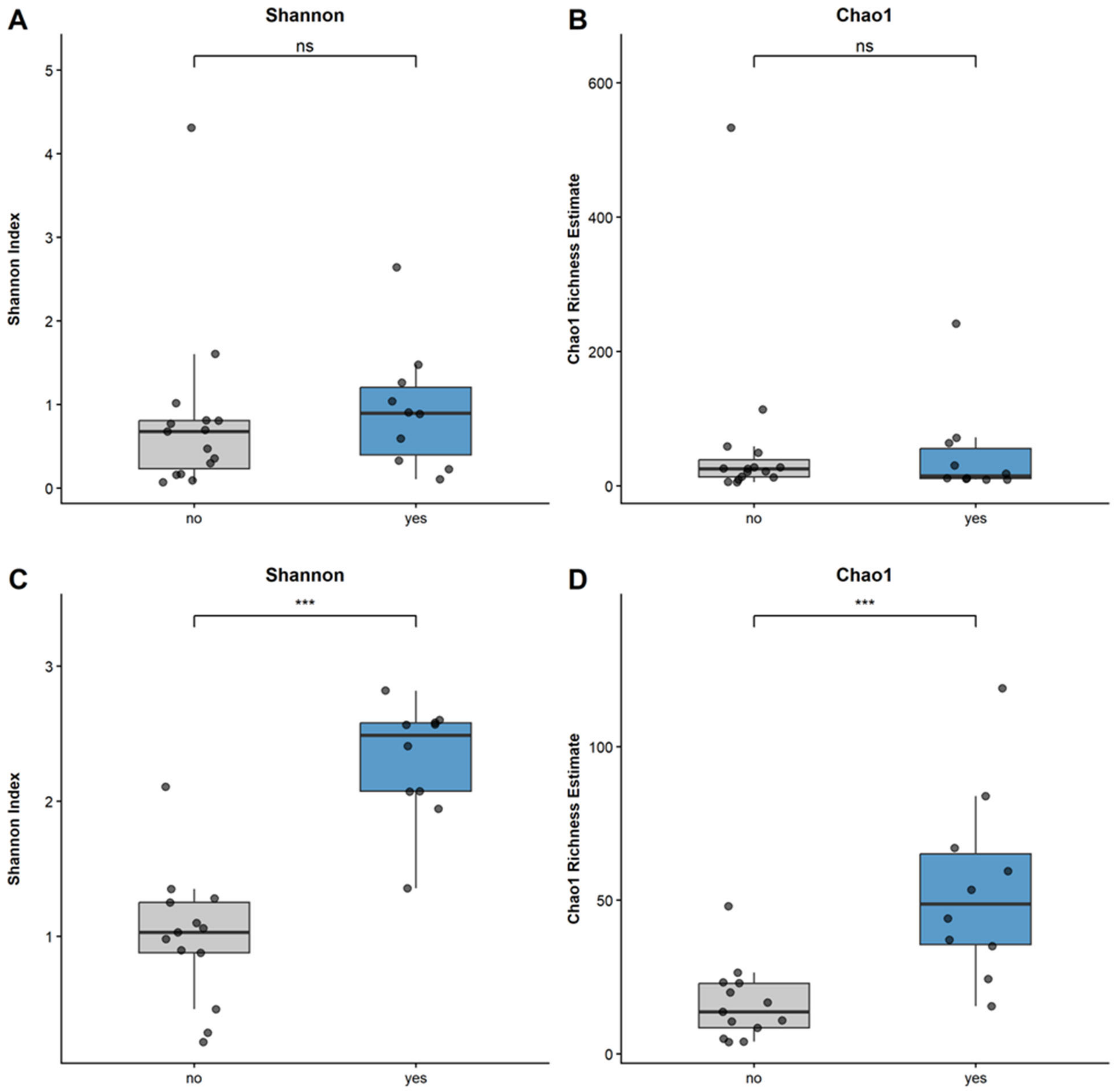

Figure S1. Alpha diversity calculated using the Shannon diversity index and the Chao1 richness estimator for both bacterial and fungal communities in subjects either using probiotics ("yes") or not ("no"). Specifically: (A) bacterial Shannon index, (B) bacterial Chao1 richness, (C) fungal Shannon index, and (D) fungal Chao1 richness. Statistical differences between groups were assessed using the Wilcoxon rank-sum test.

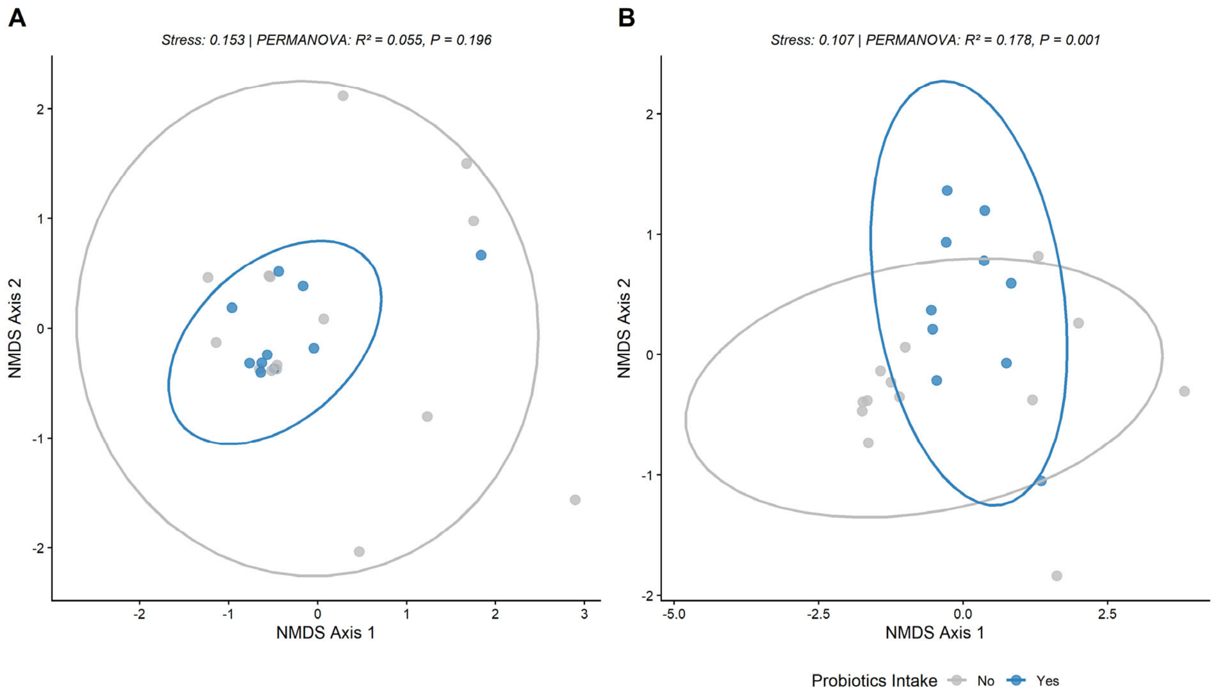

Figure S2. Beta diversity visualized using a Non-metric Multidimensional Scaling (NMDS) plot based on Bray–Curtis dissimilarity in subjects either using probiotics ("yes") or not ("no"). Panel (A) shows bacterial communities, and panel (B) shows fungal communities. Statistical differences between groups were assessed using the PERMANOVA test.

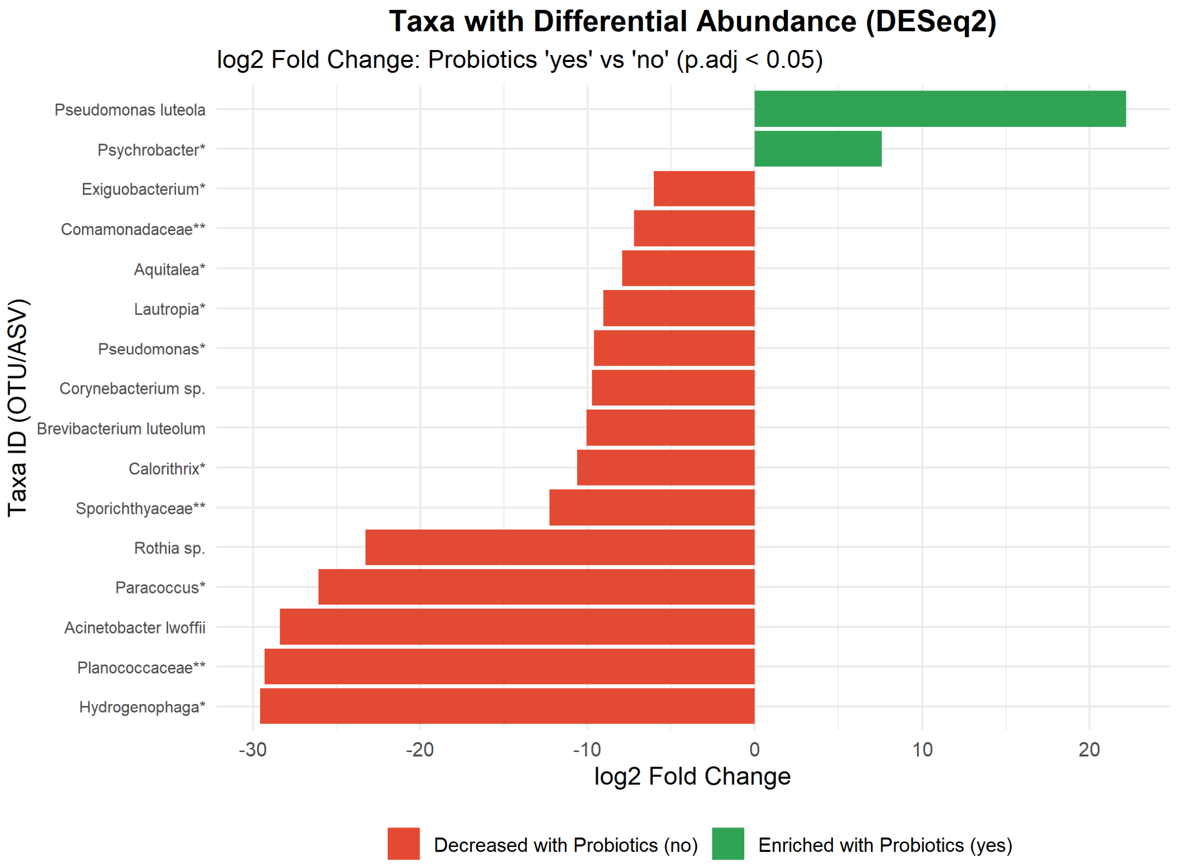

Figure S3. Differential abundance (DESeq2) of bacterial taxa between probiotic users ("yes") and non-users ("no"), showing taxa significantly enriched (green) or decreased (red) with probiotic use (p<0.05).

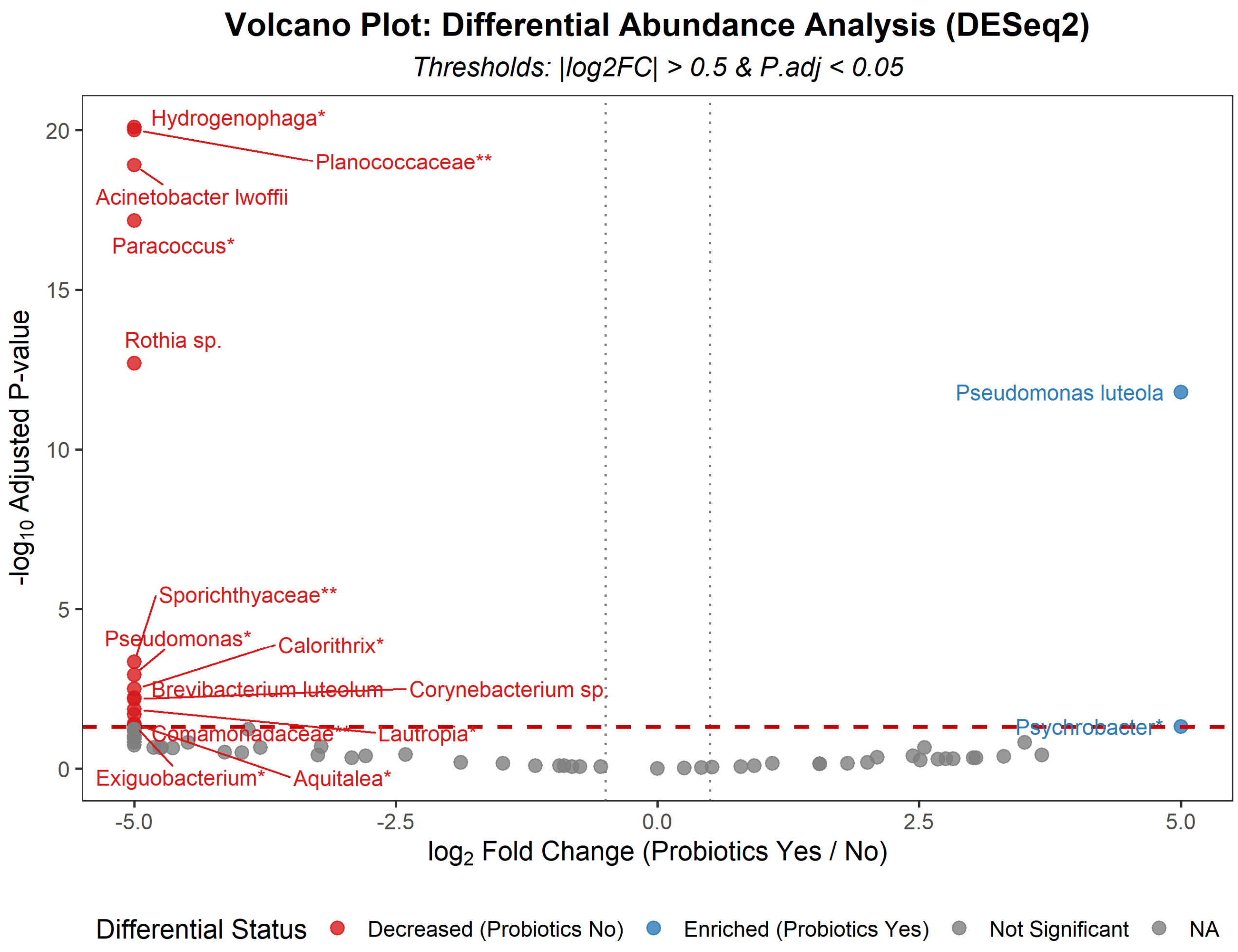

Figure S4. Volcano plot of bacterial taxa differentially abundant between probiotic users and non-users (p<0.05).

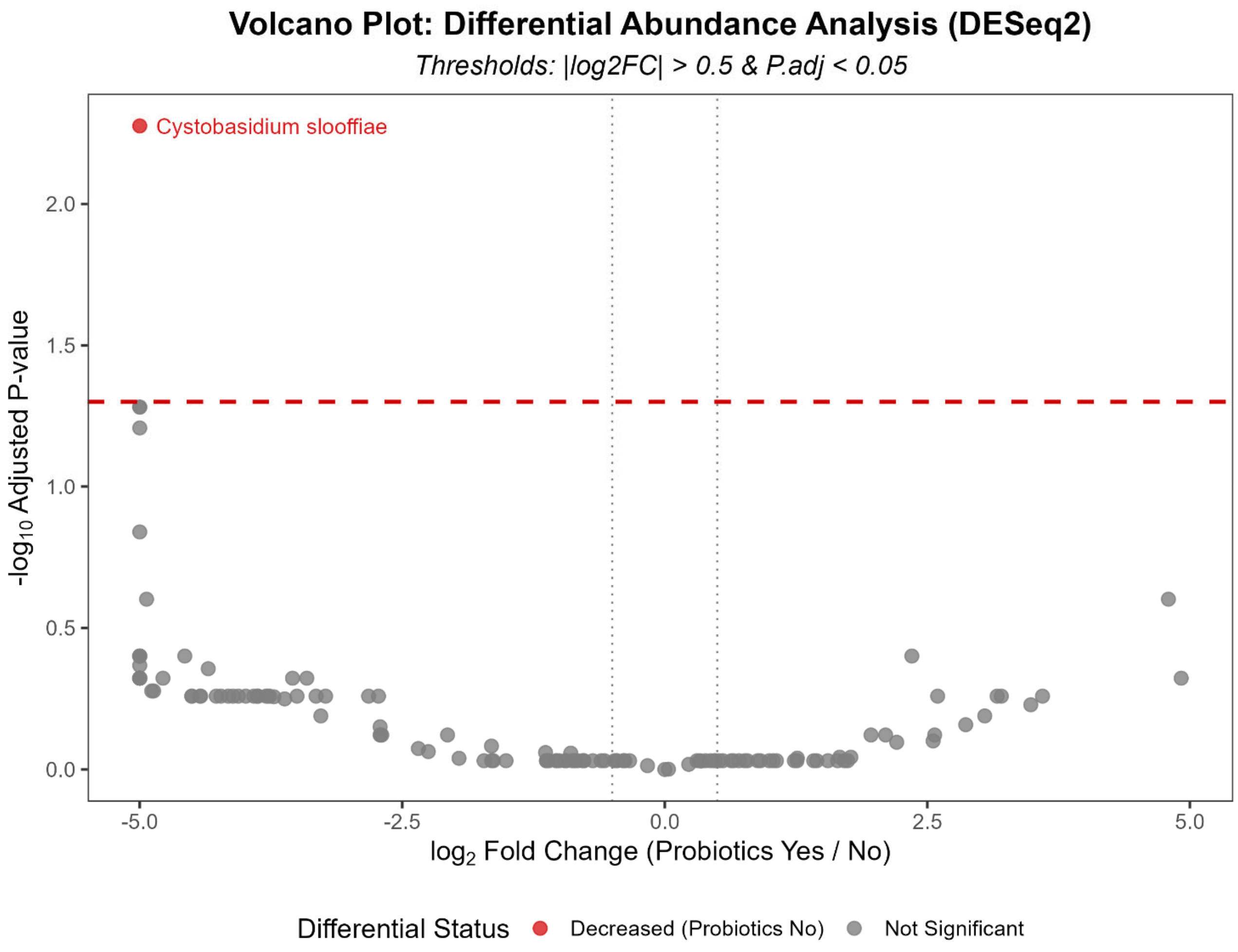

Figure S5. Volcano plot of fungal taxa differentially abundant between probiotic users and non-users (p<0.05).

References

1. Berlau, J.; Aucken, H.; Malnick, H.; Pitt, T. Distribution of Acinetobacter Species on Skin of Healthy Humans. *European Journal of Clinical Microbiology and Infectious Diseases* **1999**, *18*, 179-183, doi:10.1007/s100960050254.
2. Patil, J.R.; Chopade, B.A. Distribution and in vitro antimicrobial susceptibility of Acinetobacter species on the skin of healthy humans. *Natl Med J India* **2001**, *14*, 204-208.
3. Sahu, K.K.; Lal, A.; Mishra, A.K.; Abraham, G.M. Aerococcus-Related Infections and their Significance: A 9-Year Retrospective Study. *J Microsc Ultrastruct* **2021**, *9*, 18-25, doi:10.4103/jmau.Jmau_61_19.
4. Veysseyre, F.; Fourcade, C.; Lavigne, J.P.; Sotto, A. Bacillus cereus infection: 57 case patients and a literature review. *Med Mal Infect* **2015**, *45*, 436-440, doi:10.1016/j.medmal.2015.09.011.
5. Abbas, S.; Figueroa-Chavez, W.; Yasmin, A.; Welch, M. A good bug gone bad? Whole genome sequence and phenotypic characterisation of a soil organism, Bacillus paralicheniformis MB647, isolated from an infected orthopaedic implant. *Access Microbiology* **2024**, doi:https://doi.org/10.1099/acmi.0.000935.v1.
6. Yokota, S.; Taniguchi, T.; Takayanagi, S. Brucella anthropi bacteremia: Persistent bacteremia with minimal symptoms. *Journal of Infection and Chemotherapy* **2025**, *31*, doi:10.1016/j.jiac.2024.102595.
7. Liu, B.; Liu, G.H.; Sengonca, C.; Schumann, P.; Ge, C.B.; Wang, J.P.; Cui, W.D.; Lin, N.Q. Bacillus solani sp. nov., isolated from rhizosphere soil of a potato field. *Int J Syst Evol Microbiol* **2015**, *65*, 4066-4071, doi:10.1099/ijsem.0.000539.
8. Archambaud, C.; Nunez, N.; da Silva, R.A.G.; Kline, K.A.; Serror, P. Enterococcus faecalis: an overlooked cell invader. *Microbiol Mol Biol Rev* **2024**, *88*, e0006924, doi:10.1128/mmbr.00069-24.
9. Pontes, J.G.d.M.; Fernandes, L.S.; dos Santos, R.V.; Tasic, L.; Fill, T.P. Virulence Factors in the Phytopathogen–Host Interactions: An Overview. *Journal of Agricultural and Food Chemistry* **2020**, *68*, 7555-7570, doi:10.1021/acs.jafc.0c02389.
10. Chaturvedi, P.; Shivaji, S. Exiguobacterium indicum sp. nov., a psychrophilic bacterium from the Hamta glacier of the Himalayan mountain ranges of India. *Int J Syst Evol Microbiol* **2006**, *56*, 2765-2770, doi:10.1099/ijs.0.64508-0.
11. Frühling, A.; Schumann, P.; Hippe, H.; Sträubler, B.; Stackebrandt, E. Exiguobacterium undae sp. nov. and Exiguobacterium antarcticum sp. nov. *Int J Syst Evol Microbiol* **2002**, *52*, 1171-1176, doi:10.1099/00207713-52-4-1171.
12. Xu, P.; Zhang, D.; Zhuo, W.; Zhou, L.; Du, Y.; Zhang, P.; Ma, L.; Wang, Y. Characterization of a Highly Virulent Klebsiella michiganensis Strain Isolated from a Preterm Infant with Sepsis. *Infect Drug Resist* **2024**, *17*, 4973-4983, doi:10.2147/idr.S481750.
13. Yang, J.; Long, H.; Hu, Y.; Feng, Y.; McNally, A.; Zong, Z. Klebsiella oxytoca Complex: Update on Taxonomy, Antimicrobial Resistance, and Virulence. *Clin Microbiol Rev* **2022**, *35*, e0000621, doi:10.1128/cmr.00006-21.
14. Boonchuay, K.; Sontigun, N.; Wongtawan, T.; Fungwithaya, P. Association of multilocus sequencing types and antimicrobial resistance profiles of methicillin-resistant Mammaliicoccus sciuri in animals in Southern Thailand. *Vet World* **2023**, *16*, 291-295, doi:10.14202/vetworld.2023.291-295.
15. Davis, C.P. Normal Flora. In *Medical Microbiology*, Baron, S., Ed.; University of Texas Medical Branch at Galveston

Copyright © 1996, The University of Texas Medical Branch at Galveston.: Galveston (TX), 1996.

16. Phulpoto, I.A.; Yu, Z.; Hu, B.; Wang, Y.; Ndayisenga, F.; Li, J.; Liang, H.; Qazi, M.A. Production and characterization of surfactin-like biosurfactant produced by novel strain Bacillus nealsonii S2MT and it's potential for oil contaminated soil remediation. *Microbial Cell Factories* **2020**, *19*, 145, doi:10.1186/s12934-020-01402-4.
17. Dutkiewicz, J.; Mackiewicz, B.; Kinga Lemieszek, M.; Golec, M.; Milanowski, J. Pantoea agglomerans: a mysterious bacterium of evil and good. Part III. Deleterious effects: infections of humans, animals and plants. *Ann Agric Environ Med* **2016**, *23*, 197-205, doi:10.5604/12321966.1203878.

18. Świątczak, J.; Kalwasińska, A.; Brzezinska, M.S. Plant growth–promoting rhizobacteria: Peribacillus frigoritolerans 2RO30 and Pseudomonas sivasensis 2RO45 for their effect on canola growth under controlled as well as natural conditions. *Frontiers in Plant Science* **2024**, *Volume 14 - 2023*, doi:10.3389/fpls.2023.1233237.
19. Oliveira, I.M.F.d.; Ng, D.Y.K.; van Baarlen, P.; Stegger, M.; Andersen, P.S.; Wells, J.M. Comparative genomics of Rothia species reveals diversity in novel biosynthetic gene clusters and ecological adaptation to different eukaryotic hosts and host niches. *Microbial Genomics* **2022**, *8*, doi:https://doi.org/10.1099/mgen.0.000854.
20. Sood, U.; Hira, P.; Singh, P.; Singh, D.N.; Lal, R. Sphingomonas. In *Bergey's Manual of Systematics of Archaea and Bacteria*; pp. 1-84.
21. Geoghegan, J.A.; Irvine, A.D.; Foster, T.J. Staphylococcus aureus and Atopic Dermatitis: A Complex and Evolving Relationship. *Trends in Microbiology* **2018**, *26*, 484-497, doi:10.1016/j.tim.2017.11.008.
22. Azimi, T.; Mirzadeh, M.; Sabour, S.; Nasser, A.; Fallah, F.; Pourmand, M.R. Coagulase-negative staphylococci (CoNS) meningitis: a narrative review of the literature from 2000 to 2020. *New Microbes New Infect* **2020**, *37*, 100755, doi:10.1016/j.nmni.2020.100755.
23. Heath, V.; Cloutman-Green, E.; Watkin, S.; Karlikowska, M.; Ready, D.; Hatcher, J.; Pearce-Smith, N.; Brown, C.; Demirjian, A. Staphylococcus capitis: Review of Its Role in Infections and Outbreaks. *Antibiotics* **2023**, *12*, 669.
24. Díez de Los Ríos, J.; Hernández-Meneses, M.; Navarro, M.; Montserrat, S.; Perissinotti, A.; Miró, J.M. Staphylococcus caprae: an emerging pathogen related to infective endocarditis. *Clin Microbiol Infect* **2023**, *29*, 1214-1216, doi:10.1016/j.cmi.2023.06.006.
25. Burke, Ó.; Zeden, M.S.; O'Gara, J.P. The pathogenicity and virulence of the opportunistic pathogen Staphylococcus epidermidis. *Virulence* **2024**, *15*, 2359483, doi:10.1080/21505594.2024.2359483.
26. Krawczyk, B.; Kur, J. Chapter 16 - Molecular Identification and Genotyping of Staphylococci: Genus, Species, Strains, Clones, Lineages, and Interspecies Exchanges. In *Pet-To-Man Travelling Staphylococci*, Savini, V., Ed.; Academic Press: 2018; pp. 199-223.
27. Irlinger, F.; Loux, V.; Bento, P.; Gibrat, J.F.; Straub, C.; Bonnarme, P.; Landaud, S.; Monnet, C. Genome sequence of Staphylococcus equorum subsp. equorum Mu2, isolated from a French smear-ripened cheese. *J Bacteriol* **2012**, *194*, 5141-5142, doi:10.1128/jb.01038-12.
28. Place, R.B.; Hiestand, D.; Gallmann, H.R.; Teuber, M. Staphylococcus equorum subsp. linens, subsp. nov., a starter culture component for surface ripened semi-hard cheeses. *Syst Appl Microbiol* **2003**, *26*, 30-37, doi:10.1078/072320203322337281.
29. Rossi, C.C.; Ahmad, F.; Giambiagi-deMarval, M. Staphylococcus haemolyticus: An updated review on nosocomial infections, antimicrobial resistance, virulence, genetic traits, and strategies for combating this emerging opportunistic pathogen. *Microbiological Research* **2024**, *282*, 127652, doi:https://doi.org/10.1016/j.micres.2024.127652.
30. Ahmed, N.H.; Baruah, F.K.; Grover, R.K. Staphylococcus hominis subsp. novobiosepticus, an emerging multidrug-resistant bacterium, as a causative agent of septicaemia in cancer patients. *Indian J Med Res* **2017**, *146*, 420-425, doi:10.4103/ijmr.IJMR_1362_15.
31. Heilbronner, S.; Foster Timothy, J. Staphylococcus lugdunensis: a Skin Commensal with Invasive Pathogenic Potential. *Clinical Microbiology Reviews* **2020**, *34*, 10.1128/cmr.00205-00220, doi:10.1128/cmr.00205-20.
32. Bush, L.; Vazquez-Pertejo, M. Infezioni stafilococciche. Available online: https://www.msdmanuals.com/it/professionale/malattie-infettive/cocchi-gram-positivi/infezioni-stafilococciche (accessed on
33. Korte-Berwanger, M.; Sakinc, T.; Kline, K.; Nielsen Hailyn, V.; Hultgren, S.; Gatermann Sören, G. Significance of the d-Serine-Deaminase and d-Serine Metabolism of Staphylococcus saprophyticus for Virulence. *Infection and Immunity* **2013**, *81*, 4525-4533, doi:10.1128/iai.00599-13.
34. Akoua-Koffi, C.; Kacou N'Douba, A.; Djaman, J.A.; Herrmann, M.; Schaumburg, F.; Niemann, S. Staphylococcus schweitzeri—An Emerging One Health Pathogen? *Microorganisms* **2022**, *10*, doi:10.3390/microorganisms10040770.

35. Nam, Y.-D.; Chung, W.-H.; Seo, M.-J.; Lim, S.-I. Draft Genome Sequence of Staphylococcus vitulinus F1028, a Strain Isolated from a Block of Fermented Soybean. *Journal of Bacteriology* **2012**, *194*, 5961-5962, doi:10.1128/jb.01332-12.
36. Naveed, M.; Jabeen, K.; Aziz, T.; Hanif, N.; Waseem, M.; Khan, A.A.; Al-Harbi, M.; Alasmari, A.F. Computational Design of a Multi Epitope Vaccine Against Staphylococcus warneri for Combatting Recurrent UTIs and Skin Infections. *Mol Biotechnol* **2025**, doi:10.1007/s12033-025-01477-7.
37. Battaglia, M.; Garrett-Sinha, L.A. Staphylococcus xylosus and Staphylococcus aureus as commensals and pathogens on murine skin. *Laboratory Animal Research* **2023**, *39*, 18, doi:10.1186/s42826-023-00169-0.
38. Marina, S.S.; Bocheva, G.S.; Kazanjieva, J.S. Severe bacterial infections of the skin: uncommon presentations. *Clinics in Dermatology* **2005**, *23*, 621-629, doi:https://doi.org/10.1016/j.clindermatol.2005.07.003.
39. Chen, Z.; Xiang, K.; Wang, K.; Liu, B. Streptococcus salivarius pneumonia-associated pneumomediastinum: a case report and literature review. *BMC Infectious Diseases* **2024**, *24*, 1238, doi:10.1186/s12879-024-10138-0.
40. Horcajada, J.P.; Edwards, F.; Fonio, S.; Montero, M.; Harris, P.; Paterson, D.L.; Laupland, K.B. Pseudomonas stutzeri bloodstream infection is a prevailing community-onset disease with important mortality rates: results from a retrospective observational study in Australia. *Infect Dis (Lond)* **2024**, *56*, 606-615, doi:10.1080/23744235.2024.2333979.
41. Trofa, D.; Gácser, A.; Nosanchuk, J.D. Candida parapsilosis, an emerging fungal pathogen. *Clin Microbiol Rev* **2008**, *21*, 606-625, doi:10.1128/cmr.00013-08.
42. Miyashita, A.; Shibata, M.; Funakoshi, H.; Tame, T.; Mori, T.; Yaguchi, T.; Ban, S.; Watanabe, A.; Horikoshi, Y. First Report of Catheter-related Bloodstream Infection Caused by Filobasidium magnum. *Pediatr Infect Dis J* **2025**, *44*, e148-e149, doi:10.1097/inf.0000000000004671.
43. García-Gutiérrez, L.; Baena Rojas, B.; Ruiz, M.; Hernández Egido, S.; Ruiz-Gaitán, A.C.; Laiz, L.; Pemán, J.; Cuétara-García, M.S.; Mellado, E.; Martin-Sanchez, P.M. Fungal burden assessment in hospital zones with different protection degrees. *Building and Environment* **2025**, *269*, 112454, doi:https://doi.org/10.1016/j.buildenv.2024.112454.
44. Zare, R.; Gams, W.; Starink-Willemse, M.; Summerbell, R. Gibellulopsis, a suitable genus for Verticillium nigrescens, and Musicillium, a new genus for V. theobromae. *Nova Hedwigia* **2007**, *85*, 463-490.
45. Kim, T.; Choi, Y.; Choi, J.S.; Park, J. A case of superficial fungal infection caused by Naganishia diffluens. *Journal of Mycology and Infection* **2022**, *27*, 14-18.
